# Sarcopenic obesity: a review

**DOI:** 10.20945/2359-4292-2024-0084

**Published:** 2024-12-02

**Authors:** Victoria Zeghbi Cochenski Borba, Tatiana Munhoz da Rocha Lemos Costa

**Affiliations:** 1 Serviço de Endocrinología e Metabologia do Hospital de Clínicas da Universidade Federal do Paraná - SEMPR, Curitiba, PR, Brasil

**Keywords:** Obesity, sarcopenia, strength, muscle mass, adiposity

## Abstract

The global increase in life expectancy has led to a concomitant rise in diagnoses of sarcopenia. At the same time, the epidemic levels of obesity have given rise to the emergence of a complex condition known as sarcopenic obesity. Characterized by the simultaneous presence of loss of muscle mass and strength along with obesity or excess body fat, sarcopenic obesity represents a concerning health condition. Contrary to prevailing assumptions, sarcopenic obesity is not exclusive to older adults, as it may also manifest in individuals with obesity and chronic diseases and in those who undergo rapid weight loss. This juxtaposition of fat accumulation and muscle depletion epitomizes a harmful combination, especially in healthy adults. A precise definition of sarcopenic obesity and an understanding of how different body composition components affect functional parameters, comorbidities, and mortality rates are crucial for grasping the full extent and significance of this condition. Despite its multifaceted nature, sarcopenic obesity is often undiagnosed and undertreated, posing a considerable challenge to healthcare systems worldwide. In this review, we explore the intricate interplay of factors contributing to the development and consequences of sarcopenic obesity and discuss newly proposed diagnostic guidelines aimed at improved screening. Enhancing awareness and understanding of sarcopenic obesity is imperative for addressing its growing prevalence and mitigating its adverse health effects.

## INTRODUCTION

Given the growing impact of obesity and the increasing aging global population, it is reasonable to be concerned with a lesser-known condition that affects the lives of millions of individuals: sarcopenic obesity.

A complex and relatively recent concept, sarcopenic obesity has emerged as a considerable health concern in the last few decades. This condition represents the intersection of two major health issues: sarcopenia and obesity. Sarcopenia is characterized by the loss of muscle mass and strength, typically associated with aging, but is also present in diverse health conditions (^1^), while obesity results from an excessive accumulation of body fat.

Although sarcopenic obesity occurs frequently in old age, it can also be found in young patients who have obesity along with disabilities, chronic diseases, or a history of bariatric surgery or prolonged and inconsistent dietary regimens and weight cycling (^2^).

Sarcopenic obesity reflects a discrepancy in which the excess accumulation of body fat coexists with the depletion of muscle mass in a delicate balance that has profound implications for an individual’s overall health. The consequences of overweight or obesity on health outcomes and mortality vary depending on age, sex, health status, and methods used to diagnose sarcopenic obesity (^3^). A recent meta-analysis has added further controversy to this topic, as it found similar mortality rates among unhealthy older patients with sarcopenia, regardless of the presence of concomitant obesity. Additional obesity may worsen the health status of patients with sarcopenia, but paradoxically above the age of 65 years, sarcopenic obesity represents a biologically earlier phase with longer life expectancy than sarcopenia without obesity (^4^).

A clear definition of sarcopenic obesity and an understanding of the role that the different components of body composition have on functional parameters, comorbidity, and mortality can clarify the extent and importance of this disease (^2^). Sarcopenic obesity is a multifaceted and often underdiagnosed condition that presents unique challenges to individuals and healthcare systems worldwide. While obesity and muscle loss (or sarcopenia) have long been studied as independent health concerns, the convergence of these two conditions presents a new frontier in the realm of public health and clinical medicine (^2^).

In this review, we explore the intricate interplay of factors contributing to the development and consequences of sarcopenic obesity and discuss its impact on the lives of those affected, from increased susceptibility to chronic diseases to diminished quality of life.

### Background

Sarcopenia was first described by Irwin Rosenberg in 1989 (^5^), while the term “sarcopenic obesity”, was later introduced by Heber and cols. in 1996 as a geriatric syndrome (^6^). This was followed by the recognition of a relationship between muscle and fat mass in older adults (^7^), and the subsequent demonstration of its clinical relevance in worsening or increasing the risk of chronic diseases and adverse outcomes, even in young patients with obesity (^8,9^).

The fact that sarcopenic obesity may be diagnosed at any age, that sarcopenia associated with obesity has more severe consequences than sarcopenia or obesity alone, and that a widely accepted definition of sarcopenic obesity was lacking prompted improved diagnostic parameters. This culminated in a joint consensus statement issued by the European Society for Clinical Nutrition and Metabolism (ESPEN) and the European Association for the Study of Obesity (EASO) that may represent the starting point for a more standardized definition of sarcopenic obesity (^10^).

### Epidemiology

The prevalence of sarcopenic obesity varies depending on the population and the methodology applied for its assessment. A Korean study found a prevalence range of 0.8%-22.3% in women and 1.3%-15.4% in men (^11^). Findings from the Dutch Lifelines cohort reported an increasing prevalence of sarcopenic obesity with aging, ranging from 1.4% in women and 0.9% in men aged 18-90 years to 16.7% in both sexes at the ages of 8089 years (^12^). In the SARCOS study, the prevalence of sarcopenic obesity was 1.6% when obesity was assessed according to body mass index (BMI) and 14.1% when it was assessed according to total body fat (^13^). A meta-analysis of 50 studies including 86,285 individuals reported a global prevalence of sarcopenic obesity of 11% in adults aged 60 years and above (^14^).

The prevalence of sarcopenic obesity varies depending on the diagnostic criteria used for defining this condition. One example is an Australian study in men, which found prevalence rates of 12.6% for sarcopenia but only 0.3% for sarcopenic obesity using the European Working Group on Sarcopenia in Older People 2 (EWGSOP2) criteria (^15^). Another study found a rate of 9.6% for sarcopenic obesity using the ESPEN/ EASO criteria, relating this diagnosis to parameters of low strength and activities of daily living (^16^). Using the ESPEN/EASO definition, three Japanese studies found a 4%-14% prevalence of sarcopenic obesity (^16^). Globally, it is estimated that sarcopenic obesity will affect between 100 and 200 million people over the next 35 years, underscoring a need for effective prevention and management strategies (^17^).

### Mechanisms of sarcopenic obesity

**Figure 1:**
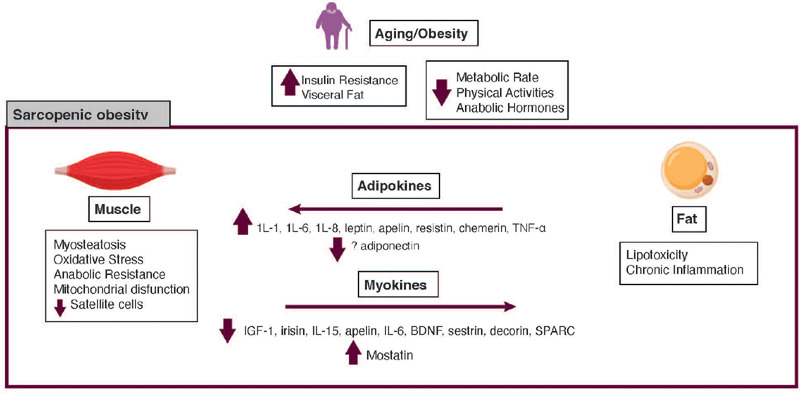
Pathogenesis of sarcopenic obesity. Abbreviations: BDNF, brain-derived neurotrophic factor; IL-, interleukin; SPARC, secreted protein acidic and rich in cysteine, TNF-α, tumor necrosis factor α.

Sarcopenia and obesity are considered multifactorial syndromes with strongly interconnected overlapping mechanisms that exacerbate each condition and lead to a vicious cycle that promotes the development of sarcopenic obesity (^18^) (Figure 1). The pathogenesis of sarcopenic obesity is complex, and its causality remains uncertain. While sarcopenic obesity and lean sarcopenia are distinct clinical entities, they share relatively similar pathophysiological mechanisms. However, they also differ in specific underlying mechanisms, such as low-grade inflammation, oxidative stress, mitochondrial dysfunction, and insulin resistance (^16,19^). Other factors related to old age and contributing to this process are physical inactivity and quantitative and qualitative malnutrition.

#### Age-related changes in body composition

Body composition undergoes substantial changes during aging, which disrupt the balance between synthesis and breakdown of muscle proteins (^18^). Aging adults lose muscle strength (quality) at a faster rate than they lose muscle mass (quantity) (^20^), culminating with an overall decline in muscle function. A contributing factor in aging muscle is its decreased regenerative capability (^21^). In homeostatic muscle, satellite cells are activated after an injury, proliferating and differentiating into myoblasts (^22^). In aging muscle, this regenerative ability gradually deteriorates, and unrepaired muscle accumulates (^19^).

#### Adiposity-related changes in body composition

Obesity is accompanied by decreased muscle mass and function (^23^). Compared with lean controls, individuals with obesity can generate greater force from a single contraction of antigravity muscles, but when the results are normalized to body mass, they show lower values than lean controls, along with increased fatigue (^24^). Individuals with obesity also have decreased muscle quality due to negative metabolic and cellular changes within the skeletal muscle.

Adiposity distribution is also an important factor in the physiopathology of sarcopenic obesity. Increases in visceral adipose tissue cause proinflammatory cytokine elevations and hormone disruption, driving sarcopenic obesity (^21^). Obesity may also stimulate the infiltration of fat into skeletal muscle, which may trigger and aggravate sarcopenic obesity (^25^). The deposition of intramyocellular lipids leads to lipotoxicity, which subsequently induces and exacerbates mitochondrial dysfunction, insulin resistance, and inflammation (^26^).

#### Increased oxidative stress and chronic inflammation

Oxidative stress is the basis of various pathologies in aging and obesity, as it induces vascular dysfunction, promotes chronic inflammation, impairs mitochondrial function, and disrupts biochemical processes (^21^). Adiposity stimulates free radical production and inflammation (^27^). Aging and obesity activate macrophages, mast cells, and T lymphocytes, leading to low-level inflammation and secretion of proinflammatory cytokines (^28^). This inflammatory response antagonizes the pro-anabolic effects of insulin growth factor-1 (IGF-1) (^19^). Proinflammatory cytokines, such as interleukin (IL)-6, IL-1, and tumor necrosis factor-α (TNF-α), play an important role in muscle homeostasis and can contribute to the pathogenesis of obesity, sarcopenia, and sarcopenic obesity by disrupting metabolic homeostasis (^18^).

#### Adipokines

In addition to their roles in energy storage and adaptive thermogenesis, white and brown adipose tissues also function as endocrine organs (^19^). These tissues can synthesize and release adipokines (*e.g.,* adiponectin, IL-1, IL-6, IL-8, leptin, apelin, resistin, chemerin, and TNF-α) (^18^), which modulate different biological processes and contribute to regulating energy expenditure, inflammation, and lipid and glucose metabolism (^29^).

Individuals with sarcopenic obesity have high levels of proinflammatory adipokines, which correlate inversely with muscle strength, prevent muscle regeneration, and promote atrophy (^30^). High leptin levels are also associated with decreased muscle quality and function (^31,32^).

Adiponectin is an insulin-sensitizing, anti-inflammatory, anti-apoptotic, and pro-angiogenic adipokine (^33^). Muscle-derived adiponectin may regulate myogenesis by influencing the proliferation and differentiation of muscle cell precursors. Serum adiponectin levels are significantly lower in patients with sarcopenia compared with those without sarcopenia, but evidence regarding adiponectin levels in individuals with sarcopenic obesity remains inconclusive (^19^).

#### Myokines

As an endocrine organ, the skeletal muscle secretes a variety of myokines through autocrine, paracrine, and endocrine processes (^18^). These myokines play different roles within the skeletal muscle itself and in other organs and tissues (^18^). Disorders in myokine secretion may be involved in the pathogenesis of age-related and metabolic diseases, including type 2 diabetes, obesity, sarcopenia, and sarcopenic obesity. Aging is associated with decreased secretion of most myokines, including IGF-1, irisin, IL-15, apelin, IL-6, brain-derived neurotrophic factor (BDNF), sestrin, decorin, and secreted protein acidic and rich in cysteine (SPARC), but increased secretion of myostatin (^34^). According to recent research, myokines may serve as diagnostic biomarkers and therapeutic targets in sarcopenia and sarcopenic obesity.

Myostatin, one of the first myokines described, is a well-known negative regulator of skeletal muscle development (^35^). It has a potential role in the development of myosteatosis (^36^) and is upregulated in obesity (^18^). The increase in myostatin levels with aging may be partially responsible for the age-related reduction in skeletal strength and muscle mass (^19^).

Irisin is a cleavage product of fibronectin type III domain-containing protein 5 (FNDC5) in skeletal muscle. It stimulates the browning of white adipose tissue and regulates thermogenesis in response to exercise under regulation by the peroxisome proliferator-activated receptor (PPAR)-γ coactivator 1a (PGC-1α) (^37^). Irisin also regulates mitochondrial function, myogenic differentiation, and metabolic homeostasis in skeletal muscle (^38^). Levels of irisin decline with age and can be increased by physical activity (^39^).

As a neurotrophin found mainly in the brain and skeletal muscle, BDNF plays a role in learning and memory (^40^). Hypothalamic BDNF is associated with regulating whole-body weight and energy homeostasis. Exercise increases the expression of BDNF in human skeletal muscle, and resistance exercise increases BDNF plasma levels (^41^). In skeletal muscle, BDNF affects myogenesis and activation of satellite cells. Levels of BDNF are low in patients with obesity and type 2 diabetes and are related to several metabolic parameters (^42^). During aging, BDNF signaling may play an essential role in regulating neuromuscular function, which may be implicated in the pathogenesis of sarcopenia and sarcopenic obesity (^19^).

In addition to the myokines mentioned above, numerous others are released in response to exercise, many of which counteract visceral obesity and play a role in myogenesis.

#### Mitochondrial dysfunction

Mitochondria play a crucial role in maintaining skeletal muscle health and overall metabolic function (^43^). In sarcopenic obesity, the balance between muscle protein synthesis and degradation is disturbed, leading to muscle wasting. This process is often accompanied by mitochondrial dysfunction, impaired energy production, increased oxidative stress, and reduced mitochondrial mass, contributing to the metabolic dysregulation observed in sarcopenic obesity (^44^).

Several mitochondrial derangements are commonly associated with aging and are worsened by obesity (^44^). Individuals with sarcopenic obesity exhibit decreased maximal oxygen uptake (VO_2_max), basal metabolic rate, and physical capacity (^45^), suggesting that sarcopenic obesity is closely associated with mitochondrial dysfunction (^44^).

### Definition of sarcopenic obesity

A systematic review published in 2022 showed considerable variability in the definition of sarcopenic obesity (^2^). Most studies define sarcopenic obesity as the coexistence of obesity and sarcopenia. However, the diagnosis of sarcopenia in some studies has been based on low muscle mass alone, without accounting for muscle strength. According to the EWGSOP2 definition, muscle strength should be the most important component in the diagnosis of sarcopenia (^46^). The systematic review also showed a lack of direct evaluation, with different muscle mass compartments being analyzed in the body composition assessment and varying normalization factors applied (^2^).

The definition of obesity also varies considerably. In the review by Donini and cols. (^2^), BMI was the most important element for defining obesity in most studies, although some also considered fat mass and waist circumference as defining factors. The assessment of fat mass requires analysis of body composition, which may be a limiting factor in some settings. Notably, waist circumference reflects excessive visceral abdominal adiposity, which may contribute directly to low muscle mass and function (^47^). To address the differences in the definition of sarcopenic obesity and emphasize the importance of muscle strength, the ESPEN/EASO consensus criteria defined sarcopenic obesity as a condition characterized by obesity (high body fat percentage) and sarcopenia (low muscle mass and function) (^10^). The consensus also established specific criteria to identify potential cases of sarcopenic obesity, regardless of the individual’s age (^10^). Since individuals with sarcopenic obesity have a relatively low muscle mass, the muscle mass reference range for the general population may be inadequate for these individuals, indicating a need to adjust muscle mass based on body mass (^48^).

### Diagnosis of sarcopenic obesity

Donini and cols. (^10^) identified the main indicator of sarcopenic obesity as either the coexistence of two conditions that could be individually assessed or the interaction between low skeletal muscle mass and high fat mass, determining a unique clinical phenotype that requires concomitant evaluation of both parameters. The quantity of skeletal muscle mass used for defining sarcopenia differs between individuals with and without obesity. In those with obesity, the relative proportions of muscle and fat mass may better define sarcopenic obesity (^2,49,50^).

The criteria and cutoff values adopted for the diagnosis of sarcopenia and obesity vary widely among published studies. The most commonly used diagnostic measurements are the appendicular skeletal muscle (ASM) divided by weight (ASM/wt) or adjusted by the squared height (ASM/h^2^) for sarcopenia and BMI for obesity. Notably, body composition parameters vary based on sex, race, and ethnicity and require appropriate reference values (^2,10^).

The ESPEN/EASO joint consensus recommends that the approach to individuals with sarcopenic obesity include screening, diagnosis, and staging (^10^). To date, only a few studies have adopted the new ESPEN/ EASO criteria for diagnosis of sarcopenic obesity, all of which have included patients with other comorbidities, including asthma (^51^), lung cancer (^52^), COVID-19 (^53^), and stroke (^54^). Two additional studies comparing the EWGSOP2 sarcopenia consensus and the ESPEN/ EASO consensus found little agreement between both in patients who underwent bariatric surgery and observed an underestimation of sarcopenic obesity in older men (^15,55^). Although the new ESPEN/EASO criteria require further endorsement, they present an opportunity to standardize the sarcopenic obesity diagnostic criteria. The sequence of investigation recommended in the ESPEN/EASO consensus statement is summarized below.

#### Screening

Screening of sarcopenic obesity should aim for maximum sensitivity. Therefore, all individuals with obesity or overweight in combination with any disease or condition related to sarcopenia (Table 1) or age older than 70 years should be suspected of having sarcopenic obesity. The starting point for the diagnosis of sarcopenic obesity is the presence of high BMI (characterizing either obesity or overweight) or high waist circumference in combination with a clinical suspicion or sign of sarcopenia.

**Table 1:** Clinical indications for screening of sarcopenic obesity

Age	>70 years
Chronic diseases (inflammatory diseases and organ failure or chronic diseases)	Heart failure Kidney failure Inflammatory bowel disease or dysfunction Liver disease (particularly NASH and liver cirrhosis) Respiratory disease Neurologic and neurodegenerative diseases Cognitive impairment Depression
Organ transplantation	
Endocrine diseases	Metabolic syndrome or diabetes Hypercortisolism Hypogonadism
Osteoarthritis	
Cancer	Active treatment
Acute diseases	Hospitalization Major trauma Immobilization or reduced mobility
Acute nutritional event	>50% reduced food intake over 2 weeks Voluntary or involuntary recent weight loss Rapid weight gain Bariatric surgery or long-duration restrictive diets
Past events	Weakness Falls Fatigue Progressive movement limitation

Abbreviation: NASH, nonalcoholic steatohepatitis.

The diagnosis of obesity may be established by a high BMI. Although BMI values vary across different ethnicities, the joint consensus recommends adopting the cutoff values proposed by the World Health Organization (WHO), *i.e.,* ≥ 27.5 kg/m^2^ for Asians and 30 kg/m^2^ for non-Asians (^10^).

The cutoff values recommended for waist circumference are ≥ 90 cm and ≥ 80 cm for Caucasian men and women, respectively, at the first level (*i.e.,* patients with a single cardiovascular risk factor) and ≥ 102 cm and ≥ 88 cm for Caucasian men and women, respectively, at the second level (*i.e.,* patients with two or more cardiovascular risk factors) and BMI of 25 to 34.9 kg/m^2^ (^56,57^). For Asian and Indians, the corresponding values are ≥ 78 cm and ≥ 72 cm, respectively, at the first level, and ≥ 90 cm and ≥ 80 cm, respectively, at the second level (^58^).

The screening criteria include age ≥ 70 years as a factor for clinical suspicion of sarcopenic obesity. However, this is not an exclusively geriatric condition, as it can also occur in middle-aged and young individuals with obesity, as well as during any acute or rapid fluctuations in body weight during weight loss treatment.

#### Diagnosis

After sarcopenic obesity confirmation screening, its diagnosis must be confirmed by demonstrating reduced functionality and altered body composition.

For measuring functionality, the parameter chosen by the ESPEN/EASO consensus is skeletal muscle strength. In the absence of a gold-standard measurement, three tests have been suggested for evaluating strength adjusted for body mass and with cutoff values adjusted for sex, age, and ethnicity. These tests include (I) the hand grip strength test, which has cutoff values of < 27 kg and < 16 kg for Caucasian men and women, respectively (^59^), and < 28 kg and < 18 kg for Asian men and women, respectively (^60^); (II) the five-time sit-to-stand chair test, which has cutoff values of ≥ 17 seconds for mixed ethnicities; and (III) the knee extension strength test, which has weight-adjusted cutoff values (strength in kg/weight in kg) of < 0.40 and < 0.31 for Caucasian men and women, respectively (^61^), and unadjusted cutoff values of < 18 kg and < 16 kg for Asian men and women, respectively (^62^).

Following the confirmation of low muscle strength, body composition should be evaluated to provide information on fat and lean body mass, preferentially obtained using dual-energy X-ray absorptiometry (DXA) or bioelectrical impedance analysis (BIA). Computed tomography imaging may be used if required for other reasons. The possibility of relatively low muscle mass should be considered, and the values should be adjusted by weight using specific cutoff values for sex, age, and ethnicity. Table 2 displays the stratified cutoff values for fat mass (^63^), skeletal muscle mass adjusted for body weight (SMM/W) (^64^), and appendicular lean mass adjusted for body weight (ALM/W) (^65^). Direct measurement of lean mass using D3-creatine dilution is the gold standard to evaluate skeletal muscle mass, but it is limited by its restricted availability and lack of wide validation. Thus, the appendicular lean mass (ALM) evaluated by DXA and corrected by body weight (ALM/W) is the preferred parameter for skeletal muscle mass evaluation, although it is affected by excessive body water and is more costly than BIA. Although BIA could also be an alternative method, it underestimates fat mass and overestimates fat-free mass in individuals with BMI > 34 kg/m^2^ (^2^). Additionally, the measurement of skeletal muscle mass using BIA is dependent on equations, may have variable cutoff points, and shows low agreement with DXA and lower sensitivity (^66,67^).

**Table 2:** Body composition threshold values for diagnosis of sarcopenic obesity

Parameter	Cutoff values
**Fat mass (%)**	**20-39 years** Caucasians Female: >39% Male: >26% Asians Female: >40% Male: >28% African-Americans Female: >38% Male: >26%
	**40-59 years** Caucasians Female: >41% Male: >29% Asians Female: >41% Male: >29% African-Americans Female: >39% Male: >27%
	**60-79 years** Caucasians Female: >43% Male: >31% Asians Female: >41% Male: >29% African-Americans Female: >41% Male: >29%
Skeletal muscle mass adjusted for body weight (SMM/W), measured using BIA	Sarcopenia Class I* Male: 31.5%-37% Female: 22.1%-27.6% Sarcopenia Class II** Male: <31.5% Female: <22.1%
Appendicular lean mass adjusted for weight (ALM/W), measured using DXA	Male: <25.7% Female: <19.4%

* Within -1 to -2 standard deviations of young adult values. ** -2 standard deviations of young adult values Abbreviations: BIA, bioimpedance analysis; DXA, dual-energy X-ray absorptiometry. Data adapted from Reference #10.

#### Staging

The purpose of sarcopenic obesity staging is to intensify treatment for individuals who need it the most. The staging is based on complications attributable to sarcopenic obesity: in stage I, no complication is present; in stage II, at least one complication related to sarcopenic obesity is present.

### Treatment

**Figure 2:**
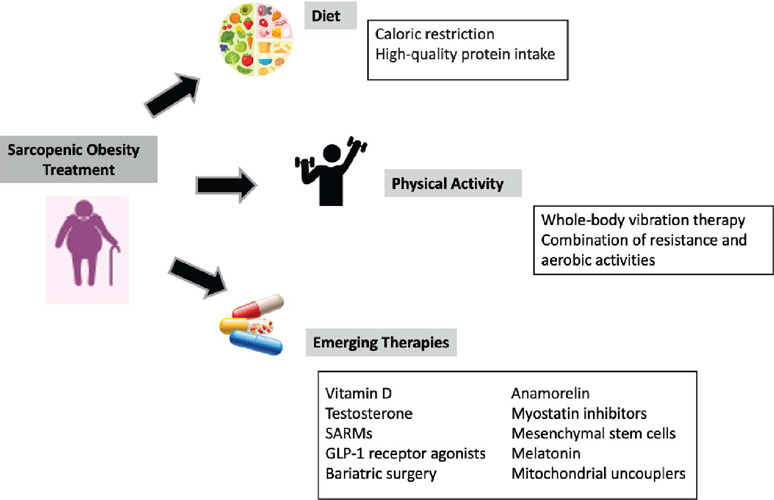
Treatment of sarcopenic obesity. Abbreviations: GLP-1, glucagon-like peptide-1; SARMs, selective androgen receptor modulators.

Due to a limited number of clinical trials focused on sarcopenic obesity treatment, the optimal therapeutic approach for this condition remains to be determined. The first-line therapy for patients with sarcopenic obesity includes exercise focused on enhancing muscle function and diet intervention (^16^). Although weight loss or exercise alone improves physical function, a combination of both enhances physical function and reduces frailty more than each intervention alone (^68^), including adults aged 65 years and older with obesity (^30^). Although several pharmacological molecules have been considered for sarcopenic obesity treatment, they are still not recommended due to a lack of strong evidence of efficacy (^69^) (Figure 2).

#### Physical exercise

Resistance, aerobic, and combination training programs reduce body fat and improve muscle function (^70^). In older individuals, exercises using mechanical overload increase levels of mammalian target of rapamycin (mTOR), inducing protein synthesis, activating satellite cells, and reducing muscle fat (^71^). Strength training in individuals with sarcopenic obesity increases protein synthesis and decreases adipose tissue and proinflammatory factors. Systematic resistance exercises lead to increased muscle fiber size, especially in fast-twitch fibers (^71^).

Aerobic activity can improve the oxidative capacity of muscle by counteracting the negative effects of intramyocellular lipids and accelerating lipolysis, resulting in increased capillary density (^30^). Aerobic training significantly reduces total body fat and visceral adipose tissue in individuals with sarcopenic obesity (^70^), helping to counteract the development of obesity (^19^). Although aerobic training is less effective than resistance training in increasing muscle mass, the combination of both leads to a more substantial effect (^36^).

Exercise recommendations for sarcopenic obesity must be individualized and include a combination of resistance and aerobic activities (^16^). For aerobic activity, 65-75% of the maximum heart rate should be reached during the exercise. Resistance training must focus on only one to two muscle groups, with the initial 8-12 repetitions at approximately 65% of the maximum strength level that the individual can generate in a single repetition. Progression should focus on using two to three muscle groups at 75% maximum intensity (^30^).

Whole-body vibration therapy involves the transmission of mechanical stimuli to activate the primary endings of muscle spindles, simulating skeletal muscle contraction and promoting neuromuscular activation. The application of vibration therapy combined with resistance training or vitamin D supplementation has shown mixed results. Although evidence in sarcopenic obesity is still limited, this alternative exercise is well-tolerated and has led to increments in skeletal muscle strength and reductions in fat mass (^72^).

#### Nutrition

The primary dietary strategy for sarcopenic obesity treatment involves caloric restriction, protein intake, and micronutrient supplementation. The quality of evidence for dietary recommendations in sarcopenic obesity is currently poor, and the existing guidelines are mainly based on expert opinion statements (^73^).

The recommendation for weight loss in older individuals with obesity remains controversial, as it is a double-edged strategy that exerts a beneficial impact by decreasing obesity-related complications but also leads to potentially negative effects related to muscle mass loss (^16^). Energy restriction with or without exercise results in the loss of approximately one-quarter of lean mass per unit weight, which could worsen sarcopenia and bone mass (^30^). A moderate energy deficit of 200-750 kcal/day with a target of 10% body weight loss in 6 months and then weight loss maintenance (^73^) is recommended. The objectives are primarily fat mass reduction and physical function enhancement. High-quality protein intake (1-1.2 g/kg/day), particularly from leucine sources, is recommended and can be combined with a calorie-restricted diet (^16^).

Conventional strategies to minimize the adverse effects of weight loss on bone metabolism include calcium and vitamin D3 supplementation (^74^). Vitamin D may improve muscle function through its bioactive metabolites, enhancing mitochondrial function and reducing oxidative stress, resulting in a possible positive impact on obesity (^16^), although consistent evidence is lacking (^75^).

#### Emerging targeted therapies

Several pharmacological therapies are emerging candidates for sarcopenic obesity treatment. Coenzyme Q10 (CoQ10) supplementation, along with other vitamins and supplements, may have a positive effect on surrogate outcomes related to physical robustness, but require further investigation (^76^). Testosterone supplementation presents a physiological rationale, particularly for increasing muscle function and strength (^77^). However, available evidence on the impact of testosterone on muscle function and mass remains conflicting. Additionally, the participation of older individuals in testosterone trials is limited due to the high risk of cardiovascular events. Currently, various scientific societies, including the American Association of Clinical Endocrinology, the Endocrine Society, and the Obesity Society, recommend against the administration of testosterone for managing obesity or sarcopenia (^16^). Similarly, in postmenopausal women, estrogen replacement therapy may be used, with generally positive outcomes on the retention of lean mass and muscle function (^77^).

Selective androgen receptor modulators (SARMs) present the advantage of selectively activating androgen receptors in bone and muscle without causing androgenic effects in other parts of the body (^77^). Although SARMs increase lean mass, no improvements in muscle strength or physical performance have been observed in older adults with sarcopenia treated with these agents (^78^). Transdermal SARMs are expected to be developed in the future.

Preclinical studies have shown that glucagon-like peptide-1 (GLP-1) receptor agonists have a beneficial effect on weight loss in sarcopenic obesity (^16^); these agents also help mitigate skeletal muscle atrophy by activating sirtuin 1 (SIRT1), which plays a crucial role in preventing age-related muscle atrophy (^79^). Weight loss induced by bariatric surgery has a consistent effect on adipose tissue mass. Its beneficial effect on muscle performance has been demonstrated in patients with obesity by comparing individuals who have undergone bariatric surgery with those who have not. Although lean mass was lower in the post-bariatric group, functional parameters were similar in both groups (^80^). Notably, performance was better in post-bariatric patients with sarcopenia compared with those who did not undergo bariatric surgery (^80^). Still, in older individuals, the safety and efficacy of bariatric surgery remain unclear, as this procedure may exacerbate sarcopenia and osteoporosis (^81^).

Anamorelin, an oral ghrelin receptor agonist, improves appetite and enhances lean mass in patients with cancer cachexia, but its role in improving muscle function or strength has not been established (^82^).

In animal models, myostatin inhibitors increase lean mass and strength, downregulate inflammatory pathways, suppress irisin, and improve insulin resistance (^83^). These agents directly reduce the expression of myostatin in muscle and adipose tissue and may be beneficial in treating patients with sarcopenic obesity (^16^).

Melatonin combined with exercise training may be a promising therapeutic approach for mitigating sarcopenic obesity. In a model of sarcopenic obesity, this combination improved the proliferation and differentiation capacity of satellite cells and mitochondria function, along with muscle mass and strength (^84^).

New therapies for sarcopenic obesity currently under investigation include mesenchymal stem cells, preclinical drugs targeting energy transduction and nutrient deposition, as well as several potential drugs, including mitochondrial uncouplers, sphingosine-1-phosphate (S1P) receptor agonists, and nuclear factor-κB (NF-ΚB) inhibitors (^76,85^). However, these therapies lack sufficient evidence of efficacy and tolerability in humans.

Overall, lifestyle modifications remain the best therapeutic approach for sarcopenic obesity, supported by the strongest and most extensive evidence. These modifications include regular aerobic and resistance exercise combined with dietary modifications with caloric restriction to reduce fat mass and increase muscle mass and function. Due to limited evidence, pharmacological therapies are not yet strongly recommended for individuals with sarcopenic obesity (^16^).

In conclusion, sarcopenic obesity was once thought to be a concern for the future but has now become prevalent in this century. It has a complex physiopathology and many uncertainties in its diagnosis, resulting in an undefined prevalence. There are currently no effective medications for sarcopenic obesity, and lifestyle modifications remain the best therapeutic approach. The new ESPEN/EASO joint consensus should improve the understanding of its epidemiology and encourage new studies in the field.
